# Effects of the Hubbard potential on the NMR shielding and optoelectronic properties of BiMnVO_5_ compound

**DOI:** 10.1038/s41598-023-33034-0

**Published:** 2023-04-10

**Authors:** H. A. Rahnamaye Aliabad, Muhammad Aamir Iqbal, F. Amiri-Shookoh, Nadia Anwar, Sunila Bakhsh, Iván D. Arellano-Ramírez

**Affiliations:** 1 Department of Physics, Hakim Sabzevari University, Sabzevar, Iran; 2 School of Materials Science and Engineering, Zhejiang University, Hangzhou, 310027 China; 3 Faculty of Physics and Nuclear Engineering, Shahrood University of Technology, P. O. Box 3619995161, Shahrood, Iran; 4 School of Materials Science and Engineering, Tsinghua University, Shaw Technical Science Building, Haidian District, Beijing, 100084 China; 5 Department of Physics, Balochistan University of Information Technology, Engineering and Management Sciences, Quetta, 87300 Pakistan; 6 Department of Physics, Universidad Tecnológica de Pereira, 660003 Pereira, Colombia

**Keywords:** Optical materials and structures, Theoretical chemistry

## Abstract

This study explores the nuclear magnetic shielding, chemical shifts, and the optoelectronic properties of the BiMnVO_5_ compound using the full-potential linearized augmented plane wave method within the generalized gradient approximation by employing the Hubbard model (GGA + U). The ^209^Bi and ^51^V chemical shifts and bandgap values of the BiMnVO_5_ compound in a triclinic crystal structure are found to be directly related to Hubbard potential. The relationship between the isotropic nuclear magnetic shielding *σ*_*iso*_ and chemical shift *δ*_*iso*_ is obtained with a slope of 1.0231 and − 0.00188 for ^209^Bi and ^51^V atoms, respectively. It is also observed that the bandgap, isotropic nuclear magnetic shielding, and chemical shifts increase with the change in Hubbard potentials (U) of 3, 4, 5, 6, and 7.

## Introduction

The BiMXO_5_ family is an analogue of the paganoite mineral BiNiAsO_5_, which was discovered in a quartz matrix (M = Cd, Ca, Mg, Mn, Pb, Ni, and X = V, As, and P). For instance, at high temperatures, BiPbXO_5_ (X = V, P) exhibits a phase change from a triclinic to a monoclinic crystal system as a result of a significant re-orientation of XO_4_. For the solid solutions BiNi_x_Co_1-x_PO_5_ and BiCoAsO_5_, transition metals also play a significant role in the complex antiferromagnetic ordering. Both BiMgVO_5_ and BiCaVO_5_ were discovered to show photoluminescence properties at room temperature in terms of their optical characteristics. Despite the fact that both VO4^–3^ and Bi^3+^ emitters have similar properties (wide emission and excitation bands associated with a large Stokes shift)^1^. The potential of Bi(III)-containing oxides as oxygen ion conductors, neon yellow pigments, catalysts, ferroelectric, ferro-elastic, and superconducting materials has been investigated, and it is reported that adding divalent or pentavalent cations in addition to Bi has the potential to increase the number of oxides that contain Bi(III). This resulted in the identification of several new compounds, including BiSr_2_V_3_O_11_, BiCa_4_V_3_O_23_, BiBa_2_V_3_O_11_, BiBa_2_V_2_PO_11_, BiMg_2_AsO_6_, BiCa_2_VO_6_, BiCd_2_VO_6_, BiCdVO_5_, Bi_3_Ca_9_V_11_O_41_, Bi_2_CaV_2_O_9_, Bi_1−2x_A_2x_VO_4-x_, BiMg_2_VO_6_, and all these compounds were made in an atmosphere of air. Some of these novel compounds showed remarkable characteristics such as nonlinear optical response, ferroelectricity and high oxygen ion conductivity^1,2^. All Mn (II) oxides were reported to be produced in the BiMnVO_5_ under vacuum or inert-gas conditions. These compounds have a number of interesting characteristics, including selective oxidation, ferroelectricity, catalysis, and vivid yellow pigments due to the presence of the stereo-active Bi^3+^ ion. Despite the synthesis of several compounds containing the magnetic M^2+^ ion, only a small amount of research has been focused on the magnetic characterization of these materials. Due to their unusual (zigzag) spin ladder architecture, the phases BiM_2_V(_P_)O_6_ (M = Cu, Mn) have drawn significant attention in terms of their magnetic properties^2,3^. Low-temperature co-fired ceramics such as BiMVO_5_ (M = Ca, Mg) compounds have attracted considerable attention because of their potential applications in microwave dielectric ceramics, ferroelectric devices, and nonlinear optoelectronics^4^. Magnetic exchange interactions of BiMPO_5_ (M = Co, Ni) compounds, on the other hand, have been observed within ferromagnetic pairs of metal ions and antiferromagnetic chains^5,6^. Isotropic exchange interactions can also be found in MFePO_5_ (M = Fe, Co, Ni, Cu)^7,8^ and Li-rechargeable batteries made of Li_3_Fe_2_, (PO_4_)_3_^9^.

For the first time, Xun et al. investigated the iso-structural compound BiMnVO_5_, using single-crystal X-ray diffraction data to determine its structural details, and reported the parameters as a = 6.912 A°, b = 6.991 A°, c = 5.354 A°, α = 108.55°, β = 95.98°, and γ = 109.73°^3^. These structures were based on MO_4_ tetrahedral with M = V, Bi_2_O_8_ groups, and MnO_6_ octahedral sharing one edge to create Mn_2_O_10_ groups. The monoclinic symmetry BiMnPO_5_ structure contains the same components. However, owing to the substantial differences in the connection of the moieties, the triclinic structure for BiMnVO_5_ cannot be viewed as a distorted variation of the monoclinic BiMnPO_5_ structure. The magnetic characterization of BiMnVO_5_ was carried out using measurements of isothermal magnetization, dc-ac magnetic susceptibility, and heat capacity. These findings show that below a transition temperature of 11.5 K, the material changes phases and becomes anti-ferromagnetic. A robust interplay of intra-dimer and inter-dimer (both intra-chain and inter-chain) contacts has been used to explain the long-range antiferromagnetic ordering. With an increase in magnetic field, inconsistency in heat capacity corresponds to the AFM transition to lower temperatures^2,3^.

The measurement of magnetic susceptibility, magnetization, and heat capacity has been reported in order to characterize the bulk magnetic properties of the dimeric chain material BiMnVO_5_^3^. This discovery offers strong support for an antiferromagnetic transition occurring at a transition temperature of around 11.5 K. Additionally, under zero field, the magnetic entropy change reaches a saturation point at 14.6 J mol^−1^ K^−1^, which is nearly equal to the entire spin entropy of Mn^2+^. Strong intra-chain and inter-chain contacts between dimers, in addition to intra-dimer interaction, are at play as long-range magnetic order develops in this chain material. Data on heat capacity at low temperatures (T < T_N_) shows that there is a gap in the spin excitations (Δ/kB ≈ 5 K). Additionally, a spin-flop transition is likely due to an abnormality in the slope of the isothermal magnetization below *T*_N_ between 30 and 40 K. This material’s weak magnetic anisotropy may be the cause of low-field spin-flop transitions and gapped spin wave excitations^3^. By combining information from measurements of isothermal magnetization and in-field heat capacity, one can create a phase diagram in the magnetic field-temperature plane.

BiMnVO_5_ is a direct bandgap semiconductor with a gap energy of 1.8 eV, and analysis of the density of states demonstrates that transitions from Mn_3*d*_/O_2*p*_ to V_3*d*_ are responsible for the optical absorption band. According to its photocatalytic performance, BiMnVO_5_ has the highest level of catalytic activity. When Methylene Blue (MB) has been exposed to visible light for 4 h, almost 98% of it degrades, from which it is established that the primary active components in the photocatalytic process are hydroxyl radicals and photo-generated holes. It can still degrade 85% of MB in 4 h when exposed to visible light after 5 cycles, and its morphology and structure are unaltered, proving that BiMnVO_5_ is a reliable photocatalyst^3^. Among the various methods, the solid-state nuclear magnetic resonance (SSNMR) technique is used for the study of structural characterization and detailed information on the interactions^10–13^. Density functional theory (DFT) calculations can be extremely powerful for the determination of nuclear magnetic resonance (NMR) spectra^14^. The chemical shifts can be computed in the plane-wave pseudopotential framework to investigate the attributes of a large range of systems^15^. The analysis of chemical shifts has been researched for several decades and has been carried out using a wide range of computational approaches^16–20^. Most NMR experiments take place in the solid or liquid state^21^.

The work is aimed at exploring the Hubbard potential (GGA + U) effects on nuclear magnetic shielding, chemical shift and optoelectronic properties of the BiMnVO_5_ compound in a triclinic crystal symmetry. The optoelectronic and NMR spectra are evaluated based on band structure, density of states, dielectric function, energy loss function, and an isotropic chemical shift, highlighting the enormous potential of these compounds for use in optoelectronic devices and sensing applications. To the best of our knowledge, this is the first study ever conducted on the BiMnVO_5_ compound. It analyzes the effects of varying the Hubbard parameter (U) on the NMR and optoelectronic properties and provides an approximate result for experimenters to fabricate devices using this material.

## Theoretical detail

In this work, all calculations were performed by solving the Kohn–Sham equations by employing a self-consistent scheme of the full potential linearized augmented plane wave (FP-LAPW) method in the framework of density functional theory (DFT)^22^ within the generalized gradient approximation along with the Hubbard potential (GGA + U) by employing the Wien2k computer code^23^. Hubbard's model of electron correlation in narrow energy bands was initially investigated by Hubbard, and he formulated this Hubbard model in 1963^24^. Since then, this method has been applied to study high-temperature superconductivity, quantum magnetism, optoelectronic properties, and charge density waves with high accuracy.

Structural data and the radii of the muffin tin spheres for BiMnVO_5_ compound that are used in this study as references are taken as a space group $P\overline{1}$ with lattice parameters *a* = 6.912 A^0^, *b* = 6.991 A^0^, *c* = 5.354 A^0^, $\alpha =$ 108.55^0^, $\beta =$ 95.98^0^, $\gamma =$ 109.73, while R_MT_ (Bi) = 2.48 a.u., R_MT_ (Mn) = 2.16 a.u., R_MT_ (V) = 1.64 a.u. and R_MT_ (O) = 1.49 a.u, respectively. The value of $k_{max}$ is used as 7, which is the cut-off for the plane wave, and R is the smallest muffin-tin radius. The value of $G_{max}$ is taken as 12, which is the value of the largest vector in charge density Fourier expansion at the plane wave cut-off. A cut-off energy of − 6 Ry caused the self-consistent computations to converge at 0.0001 Ry.

## Results and discussion

The BiMnVO_5_ compound crystallizes in the space group $P\overline{1}$ and all atoms are located on Wyckoff positions 2i^25^. The analysis of the NMR study includes the computation of nuclear magnetic shielding, isotropic chemical shift, density of states, and electronic band structure analysis. The optical properties are also deeply investigated to analyze the potential of these materials in optoelectronic devices.

### Nuclear magnetic shielding

NMR spectroscopy is an effective method for acquiring crucial information about molecular systems in chemistry and biochemistry. The nuclear magnetic shielding for BiMnVO_5_ within the GGA + U model has been computed, first familiarized by Anisimov et al.^26^ and further established by Dudarev et al.^27^. A Hubbard model is adopted to correct the self-interaction error using the following relationship:

1 $$E_{tot} = E_{DFT} + \frac{U - J}{2}\mathop \sum \limits_{\sigma } n_{m,\sigma } - n_{m,\sigma }^{2}$$

where n, m, and $\sigma$ are the atomic-orbital occupation number, the orbital momentum, and the spin index, respectively. Also, U and J show the on-site Coulomb repulsion and the exchange interaction, respectively. The exchange interaction may be incorporated into the Coulomb term to define the effective Hubbard U as $U_{eff} = U - J$^24,26^. The Hubbard potentials utilized in this work are $U_{eff} = U - J = 3, 4, 5, 6\,and\,7$. The shielding tensor ($\overline{\sigma })$, can be described by three parameters ($\sigma_{xx}$, $\sigma_{yy}$ and $\sigma_{zz}$), from which the isotropic chemical shielding $\left( {\sigma_{iso} } \right)$, can be obtained as follows^28,29^:

2 $$\sigma_{iso} = \frac{{\sigma_{xx} + \sigma_{yy} + \sigma_{zz} }}{3}$$

The three primary components of the shielding tensor ($\sigma_{xx}$, $\sigma_{yy}$ and $\sigma_{zz}$) can be defined by the Haeberlen, Mehring, and Spiess convention^30–32^. The principal components are defined in the sequence $|\sigma_{zz} - \sigma_{iso} | \ge |\sigma_{yy} - \sigma_{iso} | \ge |\sigma_{xx} - \sigma_{iso} |$.

The chemical shift values $\left( {\delta_{iso} } \right)$ of the magnetic shielding of a reference sample and of an unknown sample can be measured as follows^29^:

3 $$\delta (ppm) = \frac{{\sigma_{ref} - \sigma }}{{1 - \sigma_{ref} }} \times 10^{6}$$

where $\sigma_{ref}$ and $\sigma$ are the magnetic shielding of the reference and the sample, respectively. In this work, the BiI_3_O_9_ and VOCl_3_ compounds are considered as ^209^Bi and ^51^V, the reference combination. The tensors of magnetic shielding along the x, y, and z directions are first calculated, and the isotropic shielding values are calculated using Eq. (2). On the other hand, the isotropic chemical shift values are evaluated by the isotropic shielding values of BiMnVO_5_ in different potentials and references compounds to the formula (Eq. 3). The calculated ^209^Bi and ^51^V chemical shielding $(\sigma_{ii} )$, isotropic chemical shift $(\delta_{iso} )$ of both the compounds by using $GGA + U\,(U = 3, 4, 5, 6, 7\,{\text{eV}})$ are presented in Tables 1 and 2, respectively.

**Table 1:** The calculated components of the chemical-shielding tensor $(\sigma_{ii} )$, ^209^Bi isotropic chemical shift $(\delta_{iso} )$ by using GGA + U (U = 3, 4, 5, 6, 7 eV) for BiMnVO_5_. (All values are in ppm).

Hubbard potentials (U)	$\sigma_{xx}$	$\sigma_{yy}$	$\sigma_{zz}$	$\sigma_{iso}^{Cal}$	$\delta_{iso}$
3	2192.52	2192.52	2817.51	2400.85	− 62,7228
4	2191.35	2191.35	2822.30	2401.67	− 62,7100
5	2189.03	2189.03	2826.60	2401.55	− 62,7119
6	2168.95	2204.92	2832.46	2402.11	− 62,7032
7	2165.08	2213.83	2837.23	2405.38	− 62,6524
BiI_3_O_9_	6718.74	6465.09	6132.72	6438.85	0

**Table 2:** The calculated components of the chemical-shielding tensor $(\sigma_{ii} )$, ^51^V isotropic chemical shift $(\delta_{iso} )$ by using GGA + U (U = 3, 4, 5, 6, 7 eV) for BiMnVO_5_.

Hubbard potentials (U)	$\sigma_{xx}$	$\sigma_{yy}$	$\sigma_{zz}$	$\sigma_{iso}^{Cal}$	$\sigma_{iso}^{Thoery}$	$\delta_{iso}$
3	− 740.14	− 740.14	− 714.13	− 731.47	–	− 609,632
4	− 711.09	− 707.57	− 689.33	− 702.66	–	− 624,986
5	− 671.86	− 671.86	− 694.15	− 679.29	–	− 637,441
6	− 648.07	− 648.07	− 674.28	− 656.81	–	− 649,422
7	− 620.61	− 620.61	− 649.73	− 630.32	–	− 663,540
VOCl3	− 1646.54	− 1658.13	− 321.41	− 1875.36	− 2267.6^a^	0

All values are in ppm.

^a^^33^.

**Figure 1:**
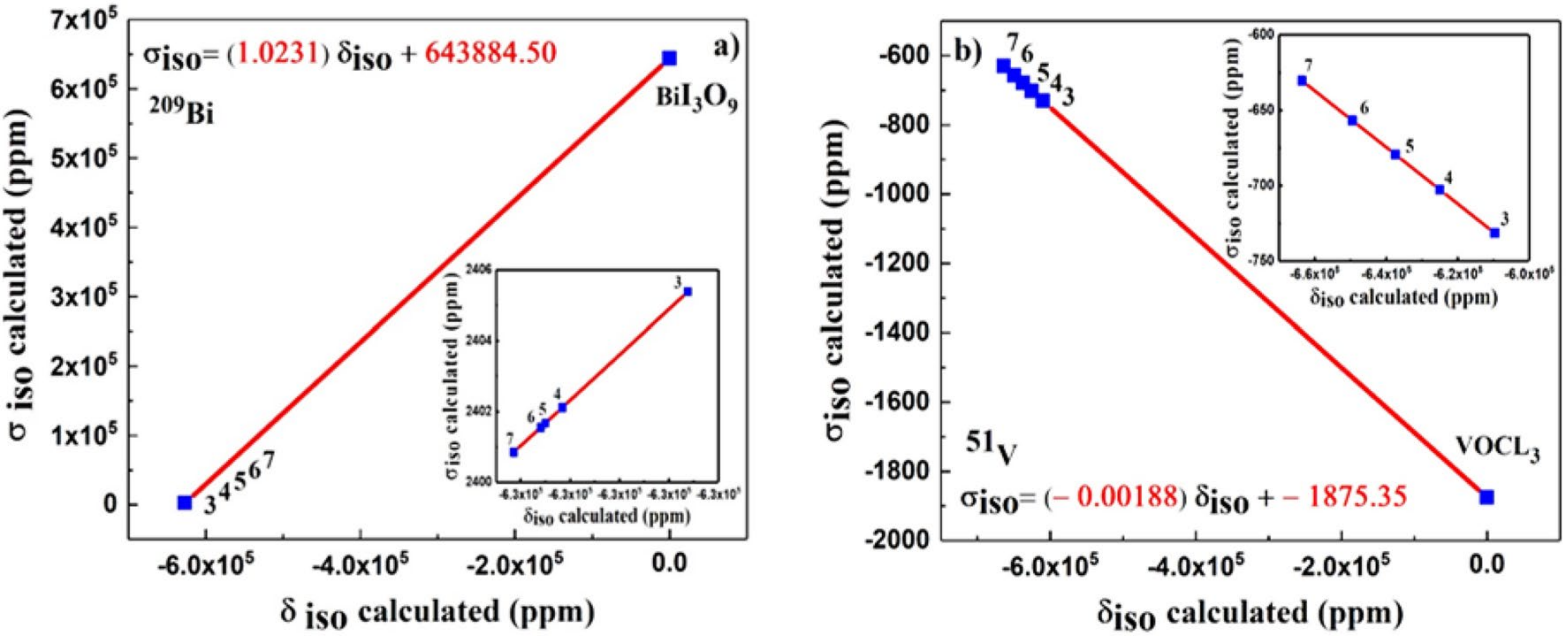
The ^209^Bi and ^51^V calculated isotropic nuclear magnetic shielding and the ^209^Bi and ^51^V measured isotropic chemical shifts that are organized in a straight line by reference compounds of BiI_3_O_9_ and VOCl_3_, (**a**) ^209^Bi σ_iso_, and (**b**) ^51^V σ_iso_. Insets in both plots show the U-dependance in a deeper detail.

The ^209^Bi and ^51^V calculated isotropic nuclear magnetic shielding and the ^209^Bi and ^51^V measured isotropic chemical shifts are organized in a straight line by reference compounds of BiI_3_O_9_ and VOCl_3_, respectively, and are shown in Fig. 1 by yielding relationships $\sigma_{iso} (Bi) = (1.0231)\delta_{iso} (Bi) + 643884.50$ and $\sigma_{iso} (V) = ( - 0.00188)\delta_{iso} (Bi) - 1875.35$.

**Figure 2:**
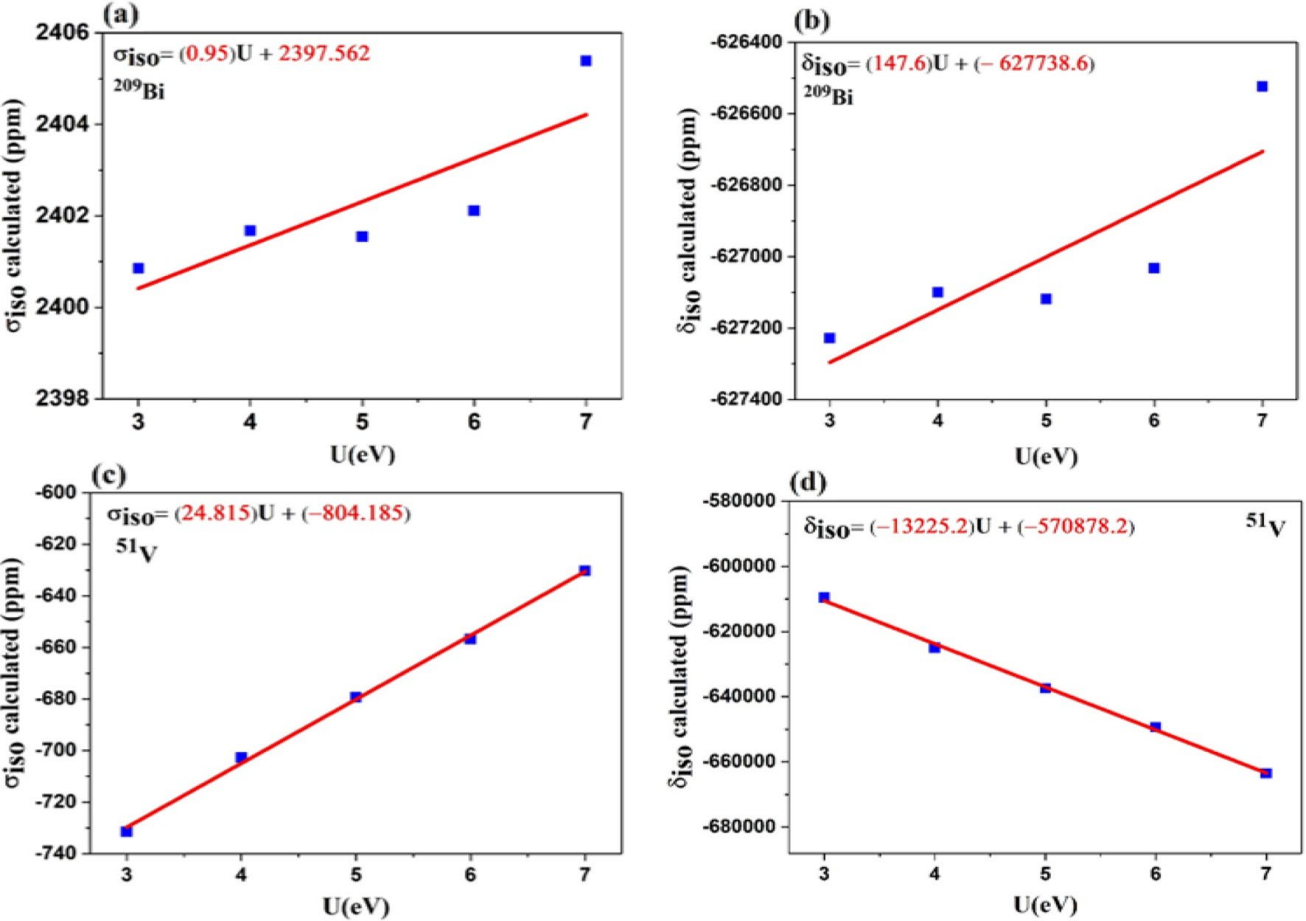
Variation of (**a**) ^209^Bi σ_iso_ values (ppm), (**b**) ^209^Bi δ_iso_ values (ppm), (**c**) ^51^V σ_iso_ values (ppm), and (**d**) ^51^V δ_iso_ values (ppm), with respect to Hubbard potential.

By increasing the $U_{eff}$ parameters, the ^209^Bi and ^51^V chemical shielding of the compounds increase from 2400.85 to 2405.38 ppm and from − 731.47 to − 630.32, respectively. As a result, isotropic chemical shift values are also increased. It seems that Hubbard’s potential plays an important role in NMR parameters. The obtained results show that the magnetic shielding tensors along the x and y directions are the same $(\sigma_{xx} = \sigma_{yy} ),$ and these values are less than the tensors along the z-direction $(\sigma_{zz} )$. Moreover, the calculated ^209^Bi and ^51^V chemical shielding and chemical shift are drawn according to Hubbard potential in Fig. 2, and they give $\sigma_{iso} (Bi) = (0.95)U + 2397.562$, $\delta_{iso} (Bi) = (147.6)U - 627738.6$, $\sigma_{iso} (V) = (24.815)U - 804.185$ and $\delta_{iso} (V) = ( - 13225.2)U - 570878.2$. The obtained results show that calculated ^209^Bi, ^51^V σ_iso_ and ^209^Bi δ_iso_ values increase with increasing Hubbard potential, while ^51^V δ_iso_ calculated values show a reverse trend with the increase in Hubbard potential. Also, the calculated magnetic shielding for the ^51^V reference compound, VOCl_3_, is in close agreement with its theoretical results^33^.

The isotropic chemical shielding for Mn and O atoms is also evaluated under the effect of varying Hubbard potential and presented for the BiMnVO_5_ compound in Table 3. The calculated chemical shielding for Mn atoms increases from 571.95 to 650.47. It can be inferred that there is an indirect link between the calculated chemical shielding for Mn atoms and the static dielectric function. The negative value of isotropic chemical shielding for ^51^V atoms shows that the external field is strengthened by the electrons.

**Table 3:** The isotropic chemical shift ($\sigma_{iso}$) for Mn and O atoms by using GGA + U (U = 3, 4, 5, 6, 7 eV) for BiMnVO_5_.

Hubbard potentials (U)	$\sigma_{iso} (Mn)$	$\sigma_{iso} (O_{1} )$	$\sigma_{iso} (O_{2} )$
3	571.95	− 112.76	− 124.19
4	600.38	− 109.39	− 120.87
5	621.10	− 106.53	− 117.11
6	637.31	− 102.02	− 113.40
7	650.47	− 98.72	− 107.90

All values are in ppm.

### Electronic properties

**Figure 3:**
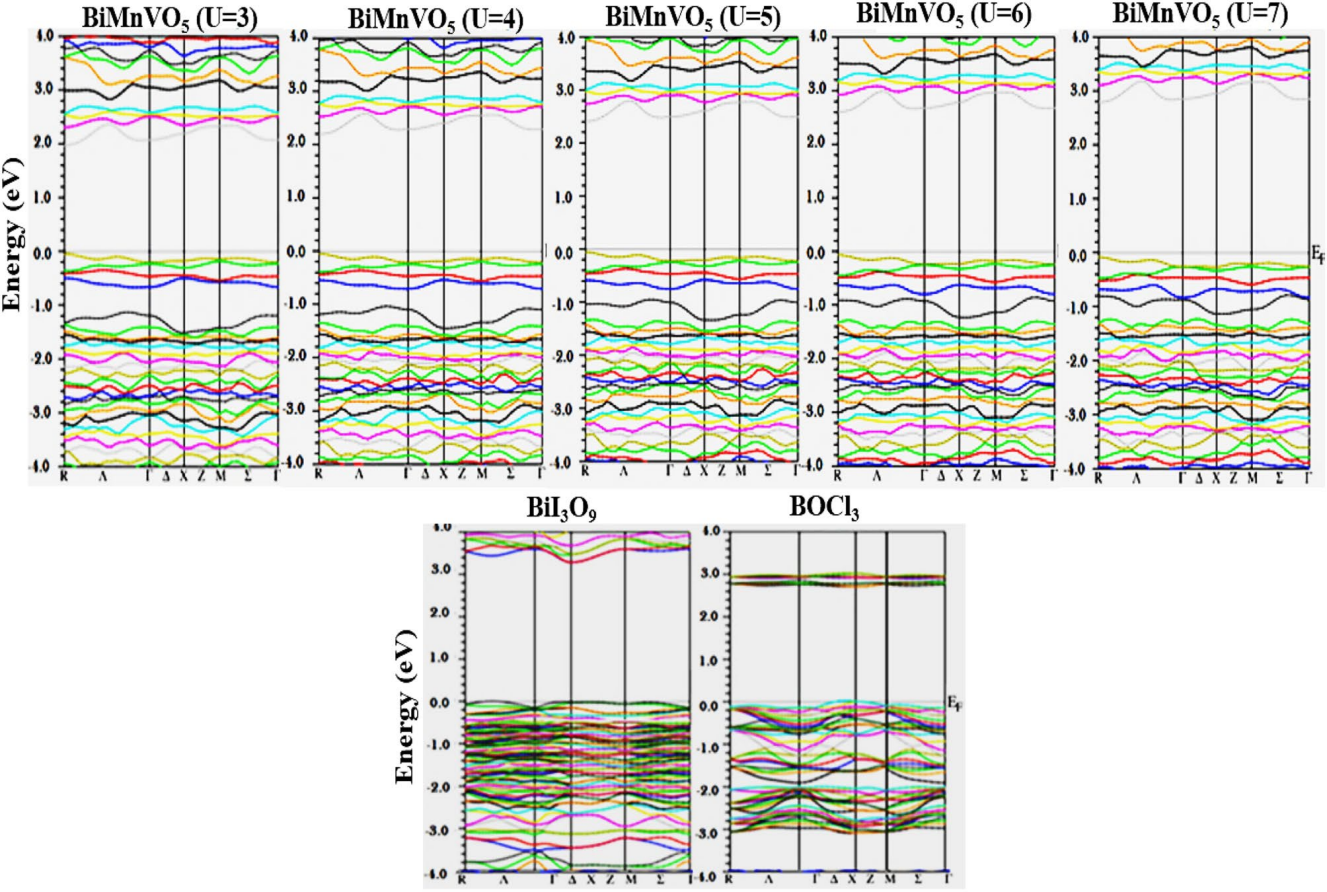
Computed band structures of BiMnVO_5_ (at U = 3, 4, 5, 6, and 7) along with the reference compounds.

In order to deeply analyze the electronic properties, the calculated band structure and density of states within the GGA + U potential with a varying value of U from 3 to 7 for the BiMnVO_5_ compound are plotted. The distance between the maximum of the valence band and the minimum of the conduction band is usually indicated as the bandgap energy (E_g_), and from Fig. 3, it can be easily deduced that bandgap values are of the indirect gap nature as the k-space vector position is located at R-Г. The energy levels are described by the quantum mechanical formalism in the band structures, and from the band structures of the BiMnVO_5_ compound, it can be inferred that the targeted material is of semiconductor nature; hence, can be potentially employed in photocatalytic applications. The generated band structures for the BiMnVO_5_ compound adopting GGA + $U\,(U = 3,4,5,6\,and\,7)$ along with the reference compounds are portrayed in Fig. 3, and bandgap values for the reference compounds are summarized in Table 4.

**Table 4:** Structural parameters and calculated bandgap values within GGA for references compounds.

Compounds	Space group	$a_{Exp.}$	$b_{Exp.}$	$c_{Exp.}$	$E_{g}^{GGA}$	$E_{g}^{Exp}$
BiI_3_O_9_	P2_1_/n^a^	8.888^a^	5.944^a^	11.221^a^	3.1	2.9^a^
VOCl_3_	P2_1_/n^b^	4.963^b^	9.140^b^	11.221^b^	2.6	2.8^c^

Lattice parameters and bandgap energy is in (Å) and (eV), respectively.

^a^^34^; ^b^^35^; ^c^^36^.

**Figure 4:**
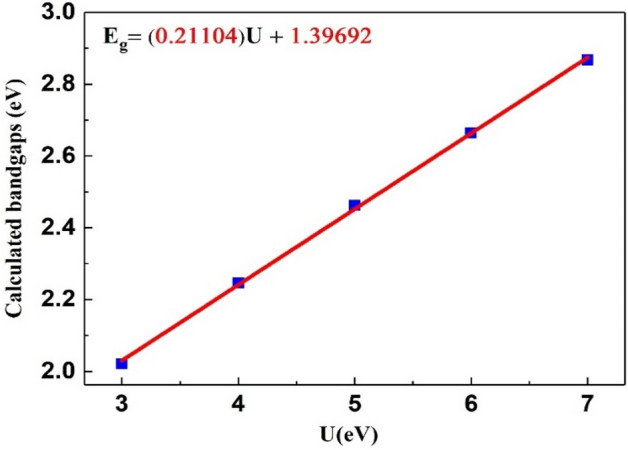
Comparison of calculated bandgap values with respect to Hubbard potential.

The calculated bandgap values are drawn according to Hubbard potential as shown in Fig. 4, and they give the band energy relation as: $E_{g} = (0.21104)U + 1.39692$. The obtained results show that the bandgap increases with increasing Hubbard potential, from a value of 3–7. It can also be observed that there is an inverse relationship between the bandgap values and the variation in the calculated isotropic chemical shift $(\delta_{iso} )$ for the ^51^V atom. The rise in bandgap may be due to the new electronic potentials generated by utilizing different U parameter values from 3 to 7. These potentials also result in a shift of conduction band electrons by lengthening the distance between the top of the valence band and the lowest of the conduction band.

**Figure 5:**
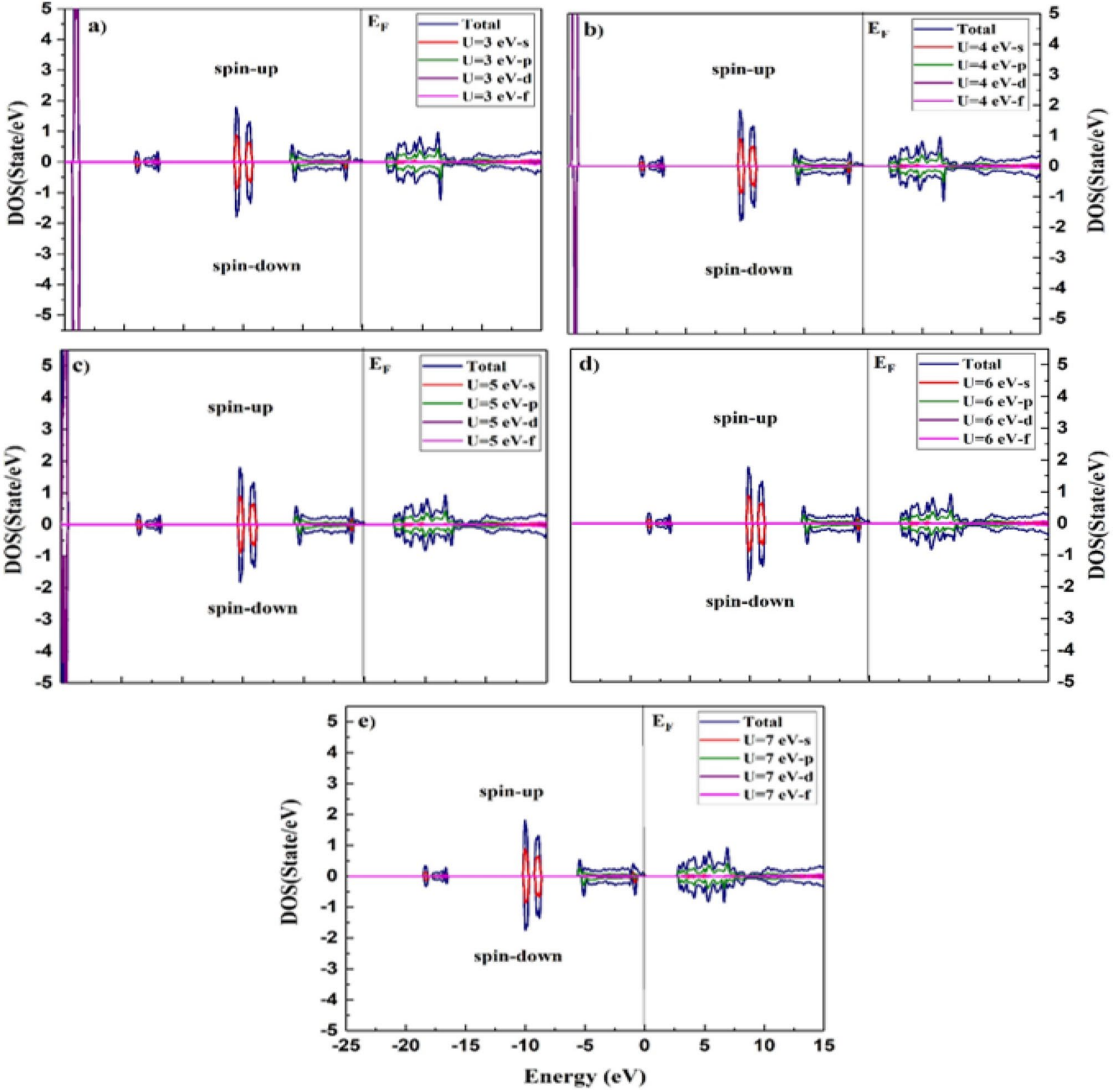
Calculated density of states of BiMnVO_5_ compound at U = 3, 4, 5, 6, and 7 for ^209^Bi by using GGA + U scheme.

**Figure 6:**
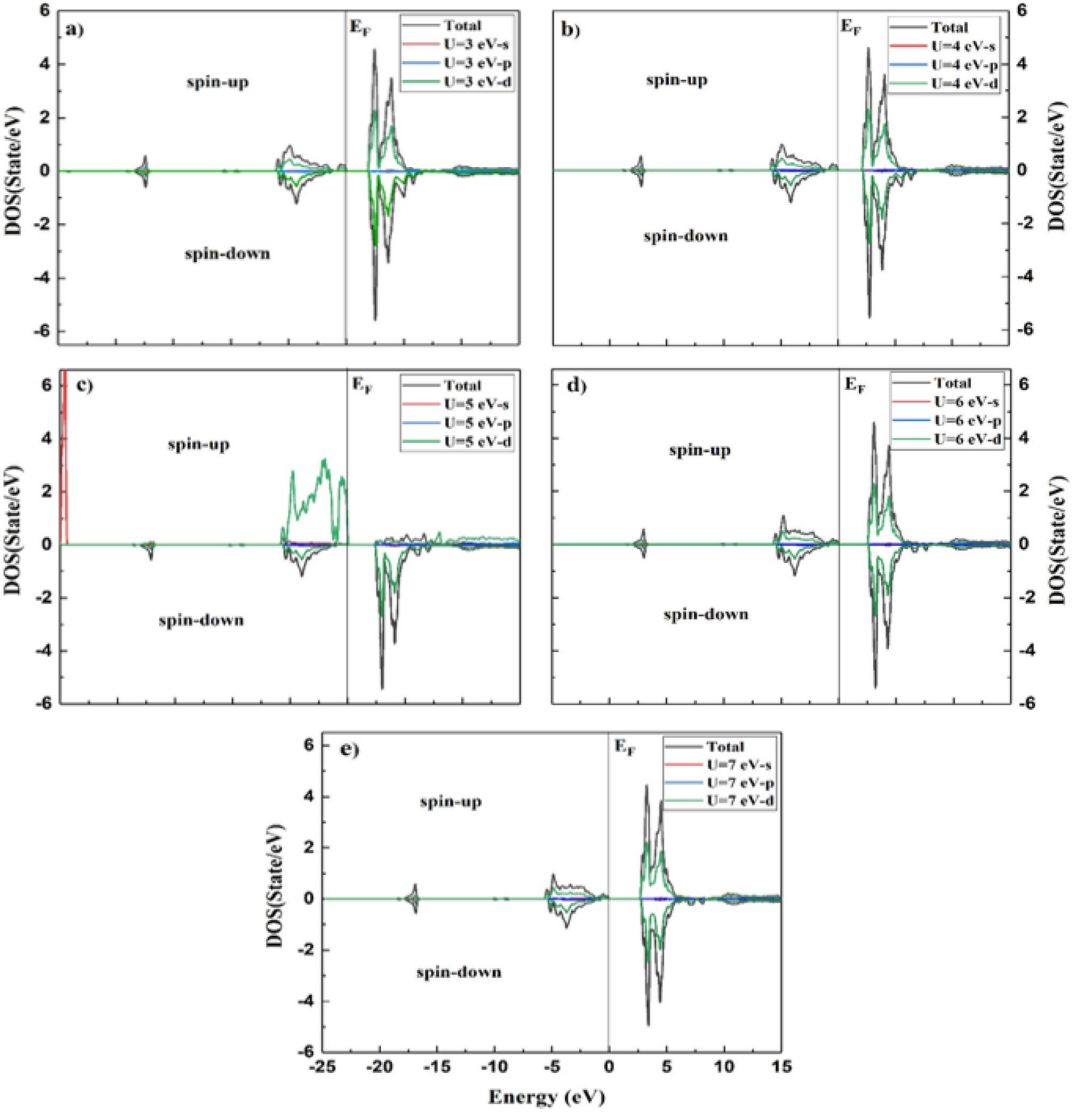
Calculated density of states of BiMnVO_5_ compound at U = 3, 4, 5, 6, and 7 for ^51^V by using GGA + U scheme.

**Figure 7:**
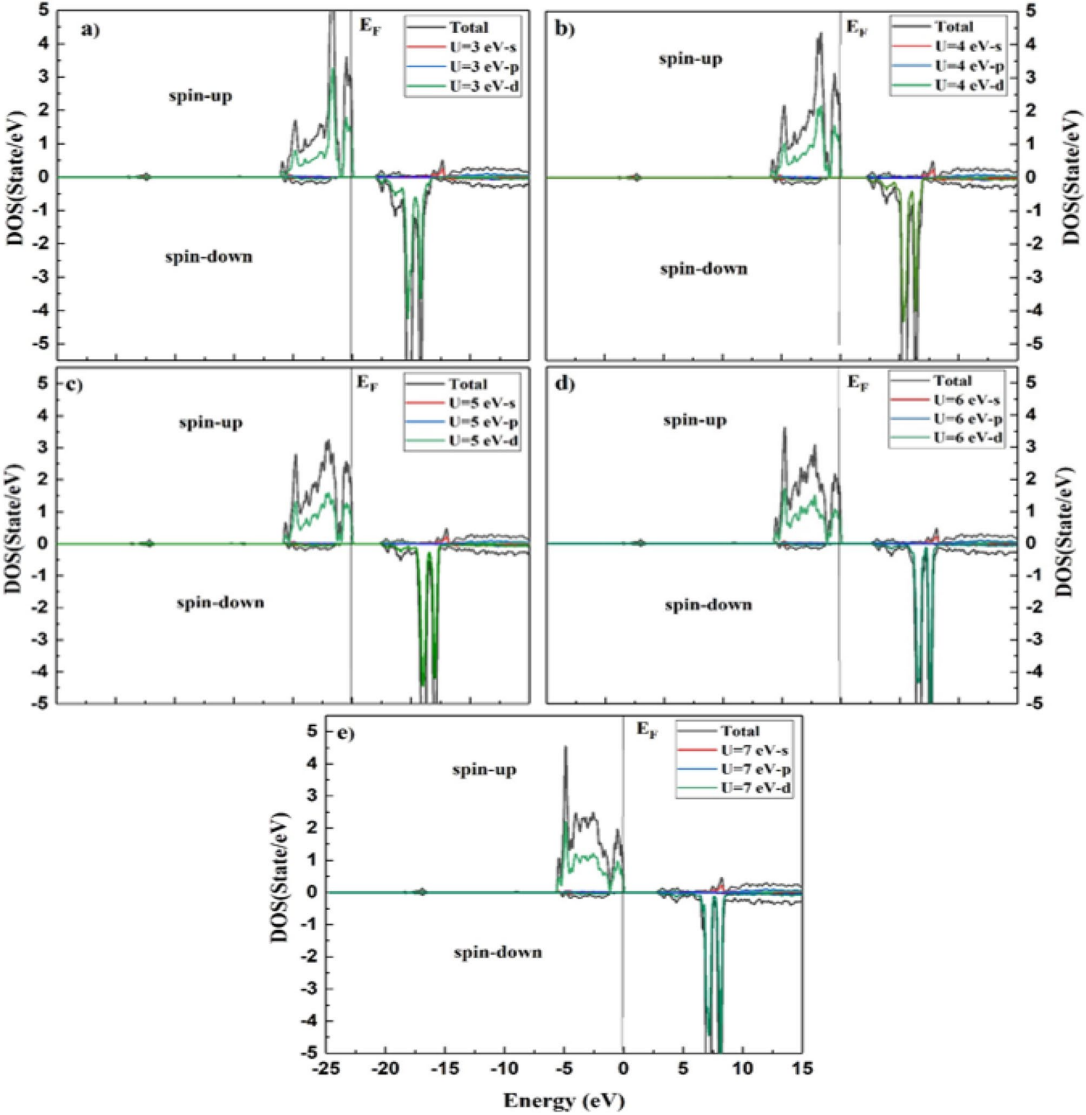
Calculated partial density of states in the spin-up and down states of Mn-s, p and d for BiMnVO_5_ compound at U = 3, 4, 5, 6, and 7.

The density of states (DOS) of a system can be described by the number of states per interval of energy at each energy level that are accessible to be busied by electrons and can be used to represent an in-depth understanding of the electronic attributes of the system. For a system, a high DOS at a specific energy level means that there are many states available for occupation, and a zero DOS means that no states can be occupied at that energy level^37^. The calculated densities of states for the ^209^Bi, ^51^V, and Mn atoms are shown in the incident energy range of − 25.0 to 15.0 eV using Hubbard values of U = 3, 4, 5, 6, and 7, as portrayed in Figs. 5, 6, and 7, respectively. The BiMnVO_5_ compound has the same behavior of DOS at all potentials. For both spin-up and spin-down states, the portion of the valence band at − 25 eV is formed of the Bi-5*d* state except for U = 6 and 7.

From Fig. 5, it can be observed that in the valence band, the Bi-6*s* state exists, whereas the conduction band is composed of the Bi-5*p* state for both spin-up and spin-down states, and these states have an important role in ^209^Bi magnetic shielding. The density of states peaks at 2.5–5.5 eV in the conduction band, generally owing to the contribution of the Bi-5*p* state. As shown in Fig. 6, for the spin-up state, the portion of the valence band at − 25 eV is primarily composed of the V-4*s* state within GGA + U at U = 5 eV. For the spin-up state, the involvement of the V-3*d* state in the valence band at 0.0–6 eV for a 5 eV potential is greater than that of other potentials. On the other hand, for the spin-up state, the top of the conduction band at 2.5–65 e is mainly influenced by the V-3*d* state in all potentials except for U = 5 eV.

The Mn atom has a considerable role in the calculation of magnetic shielding data. Therefore, the PDOS of Mn-s, Mn-p, and Mn-d states for the BiMnVO_5_ compound is perused by GGA + $U\,(U = 3,4,5,6\,and\,7)$ as portrayed in Fig. 7. For the spin-up state, the valence band at − 5.0 to 0.0 eV is primarily composed of the Mn-3*d* state, whereas for the spin-down state, these states are located at 2–7 in the conduction band. On the other hand, by moving the value of U from 3 to 7 eV, the intensity of Mn-3*d* peaks in the valence and conduction bands starts to decrease. In the spin-down state, the intensity of Mn-3*d* peaks in the conduction band is very high, and by increasing $U_{eff}$ parameters, the intensity of these peaks decreases. We found that the Mn-3*d*, Bi-*p*, Bi-*d*, and V-3*d* states have a dominating effect on the isotropic magnetic shielding value of the BiMnVO_5_ compound.

### Optical properties

For the calculation of the optical spectra, following relations are used^38,39^:

4 $$\varepsilon_{1} (\omega ) = \delta_{\alpha \beta } + \frac{2}{\pi }P\int_{0}^{\infty } {\frac{{\omega^{\prime}{\text{Im}} \varepsilon_{\alpha \beta } (\omega^{\prime})}}{{(\omega^{\prime})^{2} - \omega^{2} }}} d\omega^{\prime}$$

5 $$\varepsilon_{2} (\omega ) = \frac{{\hbar^{2} e^{2} }}{{\pi m^{2} \omega^{2} }}\sum\limits_{c,v} {\int {d{\mathbf{k}}\left\langle {c{}_{k}} \right|} } p^{\alpha } \left| {v_{k} } \right\rangle \left\langle {v_{k} } \right|p^{\beta } \left| {c_{k} } \right\rangle \times \delta (\varepsilon_{ck} - \varepsilon_{vk} - \omega )$$

6 $$L(\omega ) = - Im\left[ {\frac{1}{\varepsilon (\omega )}} \right] = \frac{{\varepsilon_{2} (\omega )}}{{\varepsilon_{1}^{2} (\omega ) + \varepsilon_{2}^{2} (\omega )}}$$

7 $$\hbar \omega_{p}^{e} = \hbar \sqrt {\frac{{ne^{2} }}{{\varepsilon_{0} m}}}$$

**Figure 8:**
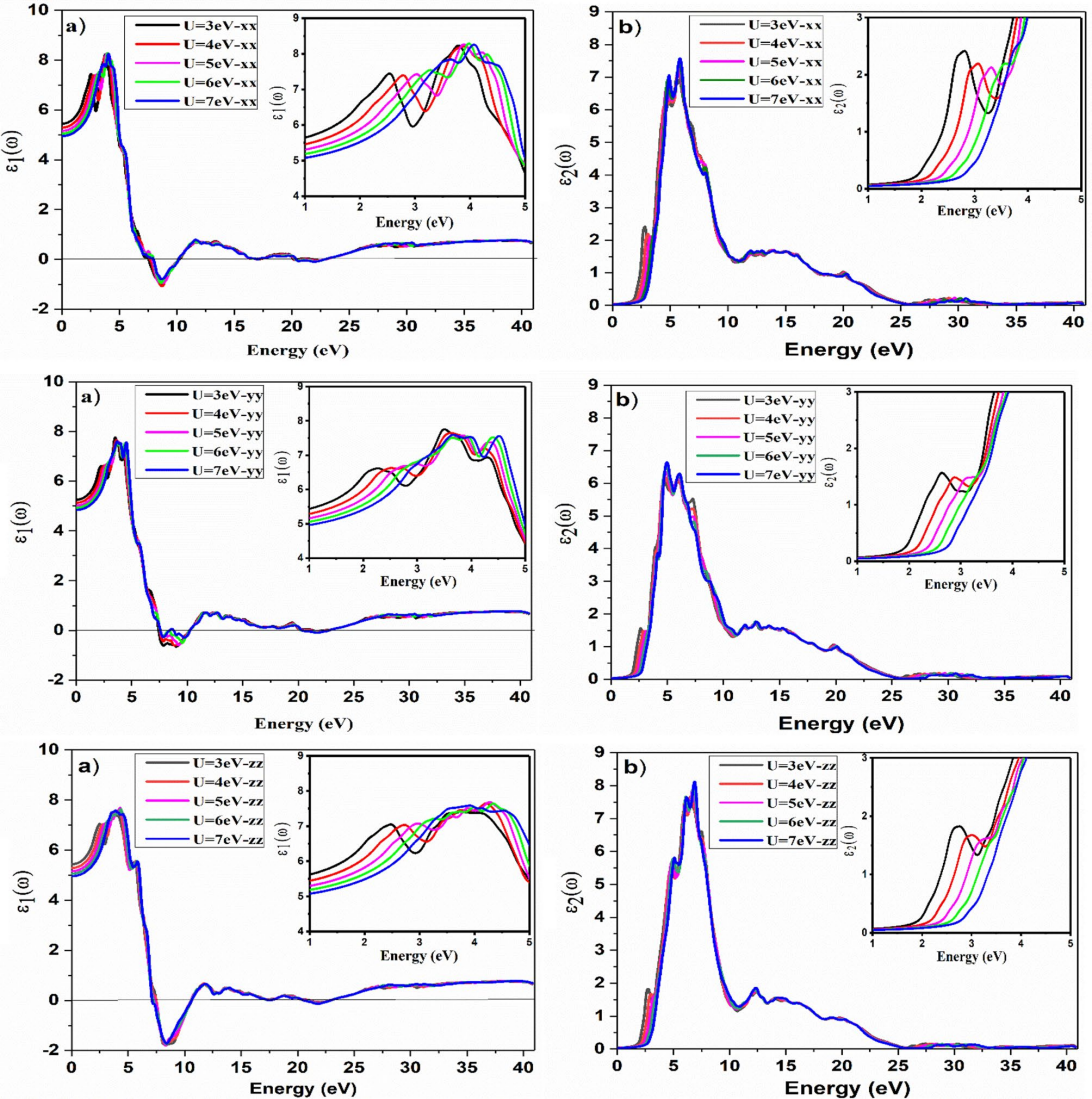
Effect of the Hubbard parameter on the optical spectra for BiMnVO_5_ compound at U = 3, 4, 5, 6, and 7, for (**a**) the real part of dielectric function and (**b**) the imaginary part of dielectric function, in the x, y, and z directions, respectively. Insets in all plots show the effect of U values in greater detail.

The real, $\varepsilon_{1} (\omega )$ and imaginary part, $\varepsilon_{2} (\omega )$ of the dielectric function are shown for the BiMnVO_5_ compound in the x, y, and z directions. From Fig. 8, it is concluded that by increasing the $U_{eff}$ parameters, the value of the static dielectric function decreases from 5.46 to 4.97 for the BiMnVO_5_ compound in the x, y, and z directions. It is clear that there is a direct relationship between the static dielectric function, $\varepsilon_{1} (0)$, and the isotropic chemical shift $(\delta_{iso} )$ for the ^51^V atom. On the other hand, the bandgap increases from 2.0208 to 2.8672, and the obtained results are observed in accordance with the Penn model^40–42^.

The maximum peaks of $\varepsilon_{1} (\omega )$ appear at 35.44, 33.18, and 37.03 for U = 6, 3, and 5 eV in the x, y, and z directions, respectively. As shown in Fig. 8b, the main peak of $\varepsilon_{2} (\omega )$ is found around 5.82, 4.99, and 6.74 in the x, y, and z directions, respectively. Indeed, these peaks show the transition from the valence band to the conduction band states. With an increase in U from 3 to 7, the main peak value increases, and this behavior confirms the variation in bandgap as well as chemical shielding for Mn atoms.

**Figure 9:**
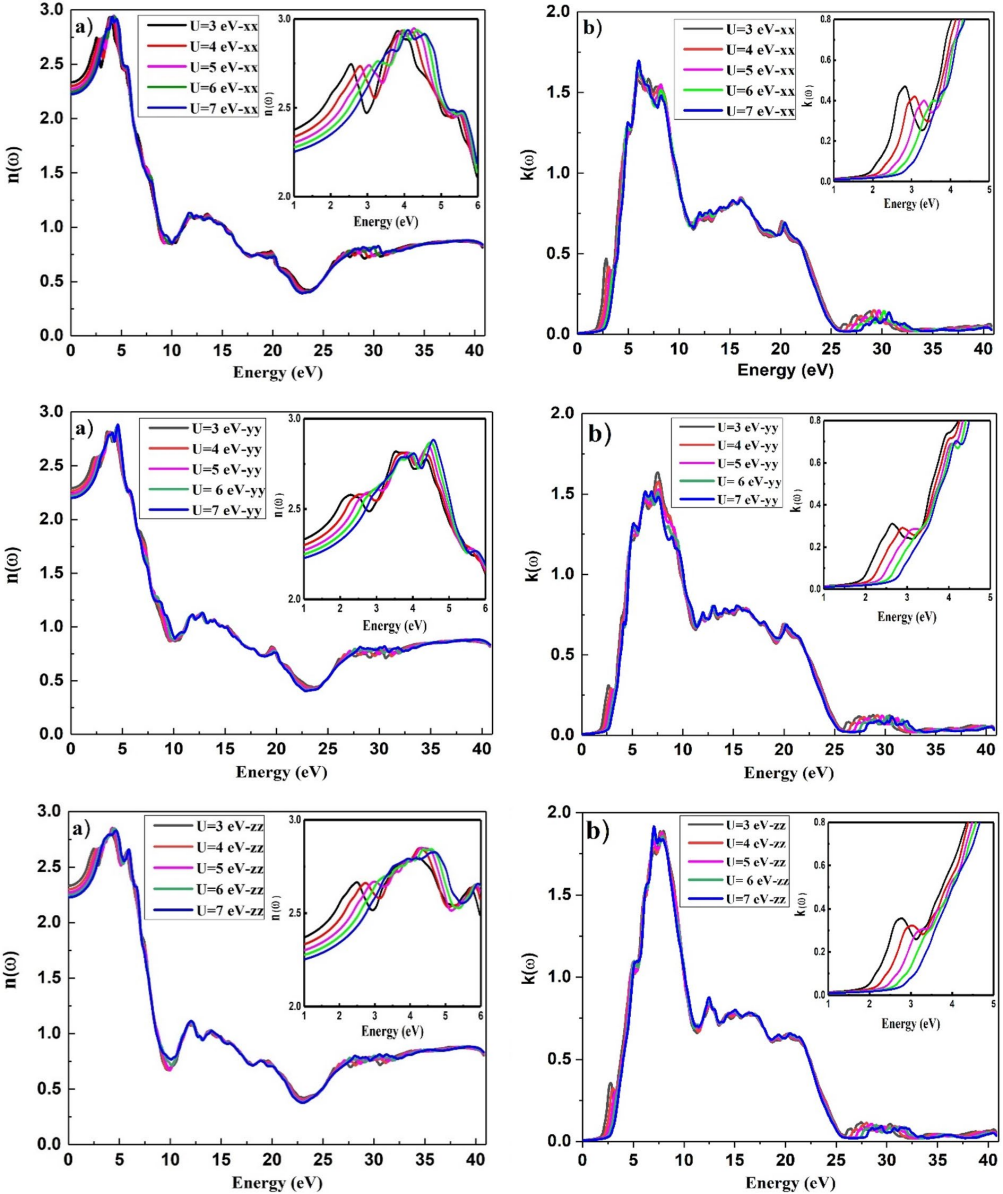
Effect of the Hubbard parameter on the refraction spectra for BiMnVO_5_ compound at U = 3, 4, 5, 6, and 7 for, (**a**) refractive index and (**b**) extinction coefficient, in the x, y and z directions, respectively. Insets in all plots show the effect of U values in greater detail.

The variation of refractive index, $n(\omega )$ and extinction coefficient, $k(\omega )$ in the x, y, and z directions is depicted in Fig. 9, from which it can be seen that the spectrum for $n(\omega )$ and $k(\omega )$ pursues the real part $\varepsilon_{1} (\omega )$ and imaginary part $\varepsilon_{2} (\omega )$ of the dielectric function, respectively. A small change $k(\omega )$ in $\varepsilon_{2} (\omega )$ may be due to medium absorption^41^, in which incident radiation is absorbed in the material. From Fig. 9a, it is easily depicted that the $n(\omega )$ initially increases and then declines firmly in a small energy interval after reaching the maximum value. Many small peaks are visible in the spectra at intermediate energies, which disappear at higher energies owing to the attraction of high energy photons by BiMnVO_5_ at different potentials. By increasing U from 3 to 7, the value of the static refraction index $n(0)$ decreases in the x, y, and z directions, and this behavior confirms the shift of the isotropic chemical shift $(\delta_{iso} )$ for the ^51^V atom in the BiMnVO_5_ compound and that it decreases with an increase in Hubbard potential. Insets in all plots of Fig. 9 show the effect of U values in greater detail on the static index of refraction.

**Figure 10:**
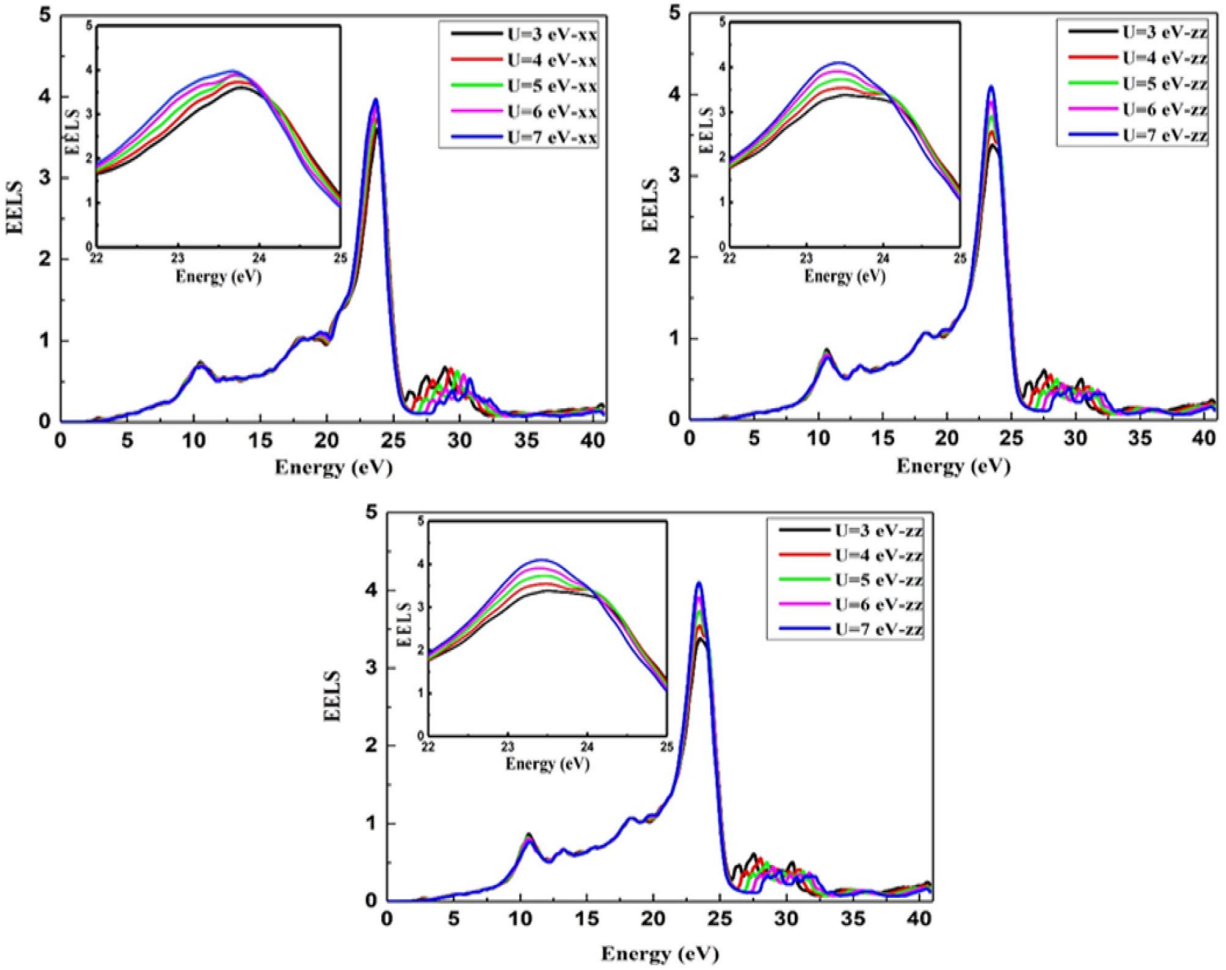
Effect of the Hubbard parameter on the energy loss spectrum for BiMnVO_5_ compound by GGA + U (U = 3, 4, 5, 6, and 7) in the x, y and z directions, respectively. Insets in all plots show the effect of U values in greater detail.

The electron energy loss spectrum (EELS) is a useful tool for investigating various material properties^43–45^. The EELS spectrum is plotted in the x, y, and z directions for the BiMnVO_5_ compound as shown in Fig. 10. The energy of the main maximum and the energy of the volume plasmon are the same. The electron plasmon energy increases by increasing the $U_{eff}$ parameters in the x, y, and z directions, and this treatment is in accordance with a variation in ^209^Bi and ^51^V σ_iso_ and ^209^Bi δ_iso_ calculated values at the Hubbard potential of U = 3, 4,5, 6, and 7. Insets in all plots of Fig. 10 show the effect of U values in greater detail on the energy loss function with an incident energy range of 22–25 eV, from which it can be seen that the electron energy loss increases as the U values increase from 3 to 7, and a maximum loss function has been observed at a high value of the Hubbard parameter.

## Conclusions

This study reports the ^209^Bi and ^51^V magnetic shielding, electronic, and optical properties of the BiMnVO_5_ compound within the GGA + U approximation by employing DFT. The obtained results show that Hubbard potential changes the shielding values, bandgap, and optical properties. The calculated ^51^V $\sigma_{iso}$ and $\delta_{iso}$ values show that there is an inverse relationship between the ^51^V NMR shielding data and bandgap values. On the other hand, it seems that there is a direct relation between the ^51^V chemical shift and the static dielectric function. The calculated PDOS patterns show that the valence band is mainly composed of the Bi-s state, whereas the conduction band is primarily composed of the Bi-p state for both spin-up and spin-down states, and these states play an important role in ^209^Bi magnetic shielding. Nevertheless, the Hubbard potential approach is successful in predicting theoretical NMR chemical shifts for ^209^Bi and ^51^V nuclei and optoelectronic properties of correlated systems.

## Data Availability

The datasets used and analyzed during the current study are available from the corresponding author (H. A. Rahnamaye Aliabad) on a reasonable request.
